# Ancient and Recent Positive Selection Transformed Opioid *cis*-Regulation in Humans

**DOI:** 10.1371/journal.pbio.0030387

**Published:** 2005-11-15

**Authors:** Matthew V Rockman, Matthew W Hahn, Nicole Soranzo, Fritz Zimprich, David B Goldstein, Gregory A Wray

**Affiliations:** **1**Department of Biology, Duke University, Durham, North Carolina, United States of America,; **2**Center for Population Biology, University of California, Davis, California, United States of America,; **3**Department of Biology, University College, London, United Kingdom,; **4**Department of Clinical Neurology, Medical University of Vienna, Vienna, Austria; **5**Institute for Genome Sciences and Policy, Duke University, Durham, North Carolina, United States of America; The Wellcome Trust Sanger InstituteUnited Kingdom

## Abstract

Changes in the *cis*-regulation of neural genes likely contributed to the evolution of our species' unique attributes, but evidence of a role for natural selection has been lacking. We found that positive natural selection altered the *cis*-regulation of human *prodynorphin,* the precursor molecule for a suite of endogenous opioids and neuropeptides with critical roles in regulating perception, behavior, and memory. Independent lines of phylogenetic and population genetic evidence support a history of selective sweeps driving the evolution of the human *prodynorphin* promoter. In experimental assays of chimpanzee–human hybrid promoters, the selected sequence increases transcriptional inducibility. The evidence for a change in the response of the brain's natural opioids to inductive stimuli points to potential human-specific characteristics favored during evolution. In addition, the pattern of linked nucleotide and microsatellite variation among and within modern human populations suggests that recent selection, subsequent to the fixation of the human-specific mutations and the peopling of the globe, has favored different *prodynorphin cis*-regulatory alleles in different parts of the world.

## Abstract 2

Strong positive selection has resulted in changes to the regulation of the human *prodynorphin* gene, with evidence for increased expression as compared with chimp. Additionally, recent selection has led to different alleles in different parts of the world.

## Introduction

Discovering the genetic changes that accompanied the origins of modern humans and pinpointing the subset of changes driven by natural selection remain central problems in evolutionary anthropology. These changes are likely to have included changes in the complement of genes, changes in the amino acid sequences of proteins, and changes in *cis*-regulation. While divergence in gene complement [1–4] and amino acid sequence [5–9] are discernable from genome sequences, functional divergence in *cis*-regulatory regions is largely invisible in sequence data. Consequently, while we now know of many uniquely human aspects of gene complement and protein sequence, we possess only a few documented examples of human-specific *cis*-regulation [10,11]. Moreover, while statistical tests for discerning the signature of positive selection in protein-coding sequences are well developed, and genomic surveys have identified many human genes showing evidence of positive selection [12,13] or diminished negative selection [14], in only a single instance has positive selection been implicated in *cis*-regulatory divergence between humans and other apes [15]. Our ignorance of *cis*-regulatory divergence is all the more remarkable considering the importance assigned to such changes in models of evolutionary novelty [16–20]. Thirty years have passed since King and Wilson [21] argued that human evolution owes more to changes in gene regulation than to changes in gene structure, and although their theoretical justifications remain strong, empirical study of human regulatory evolution has not kept pace [22].

An understanding of the genetic basis for human traits necessarily focuses on the evolution of the brain. Inquiries into human brain-specific gene regulation have relied on phenotypic analyses, particularly microarray measurements of gene expression in post-mortem human and ape brain tissue. These analyses document extensive differences in gene expression between humans and other great apes [23–26], but the genetic basis of such differences—if any—remains unknown. Moreover, a specific class of change in gene regulation, change in transcriptional inducibility, is invisible to studies of post-mortem tissues. Our species is distinguished by the ability to respond to and manipulate environmental cues; the responsiveness of genes to such cues, and not merely their constitutive activity, may play a role in human evolution.

Given the difficulties associated with identifying DNA sequence changes responsible for changes in inducibility, we focused on a candidate region whose role in inducibility in humans has already been demonstrated. The a priori designation of a functional regulatory element allows us to apply the tools of molecular evolution developed for protein sequences—specifically, rate comparisons among classes of nucleotide sites [20]. Of necessity, we also investigated the functional consequences of the species differences experimentally; we used transient transfection of promoter-reporter constructs into cultured human cells, a method with a record of success in identifying functional variation within our species [27–30].

We studied the *cis*-regulatory evolution of the opioid neuropeptide precursor prodynorphin (*PDYN*) (OMIM 131340). The opioid neuropeptides (endorphins) are the endogenous ligands for the opiate receptors. They mediate the anticipation and experience of pain [31,32], they influence behaviors including social attachment and bonding [32,33], and they affect learning and memory [32,34]. One of *PDYN'*s products is dynorphin, a peptide whose pharmacological analogs specifically affect perception [35]. A 68 base-pair (bp) tandem repeat polymorphism in the human *PDYN* promoter, 1,250 bp upstream from the start of transcription, influences the inducibility of the gene [36], and association studies have tentatively implicated the polymorphism in schizophrenia [37], cocaine addiction [38], and epilepsy [39]. These genetic associations are supported by physiological associations between *PDYN* expression and each of the phenotypes [40–42].

## Results

### Phylogenetic Evidence: Accelerated Evolution of a Functional Element in the Human *PDYN* Promoter

**Figure 1:**
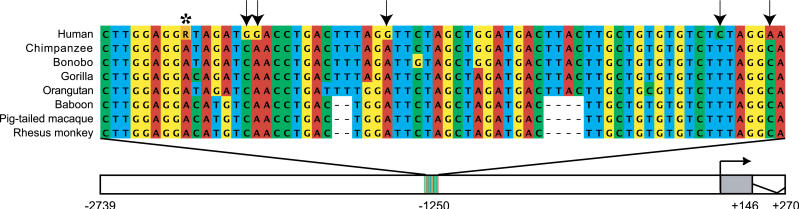
Divergence of the 68-bp Element in Humans Arrows indicate five differences fixed on the human lineage. The asterisk indicates a site that varies among human repeats. In the sample of 74 human haplotypes, all one-repeat and most two-repeat alleles bear G at this site. Complete haplotype data are given in Table S1. Below, schematic of the study region showing the position of the element and the non-coding first exon with respect to the start of transcription.

To understand the evolutionary basis for the functional variation, we sequenced 3 kilobases (kb) of *PDYN* regulatory DNA from 74 human chromosomes and 32 chromosomes from seven species of non-human primates, experimentally determining haplotypic phase by cloning each allele. The non-human primates bear a single copy of the 68-bp regulatory element, and the pattern of substitutions implies that the duplication of the element is specific to the human lineage. All human copies of the element carry five substitutions that differentiate them from the sequence inferred for the last common ancestor of humans and chimpanzees. A sixth difference is variable among repeats in some human haplotypes (Figure 1).

The five substitutions fixed on the human lineage are dramatically more than expected for 68 bp of neutrally evolving sequence. Under a model of spatially random mutation, the expected number of substitutions is fewer than 0.5, and the observed number is extremely improbable (Poisson *p* < 0.0001), whether we calculate the expectation from the density of substitutions across the *PDYN* promoter, the average divergence between human and chimpanzee on a chromosomal scale [43], or the estimated great ape substitution rate [44].

The elevated number of substitutions may be due to locally elevated mutation rate or to positive selection increasing the probability of fixation of new mutations. If the local mutation rate is intrinsically elevated, other species should also exhibit rapid evolution in the 68-bp region. We therefore tested a molecular clock, using phylogenetic likelihood ratio tests [45], to ask whether the 68-bp region is evolving rapidly due to an elevated mutation rate. The evolution of the 68-bp element is significantly accelerated exclusively along the branch leading to humans from our last common ancestor with chimpanzees (*p* = 0.005). The other branches of the evolutionary tree show no departure from rate constancy (*p* = 0.657), and the remainder of the promoter region and the coding sequence of *PDYN* also show no acceleration (Table 1). To control for possible lineage specific rate variation, we applied the relative ratio test [46], which allows for lineage-specific rates and for DNA region-specific rates, and tests for lineage-by-region interactions. Here again, the human 68-bp repeat exhibits a significant departure from the neutral expectation (*p* = 0.001 for proportionality to the rest of the promoter, *p* = 0.015 for proportionality to the coding sequence; Table 2); the remaining lineages and regions exhibit no such departures. The phylogenetic data imply that the rapid evolution of the human 68-bp element is due to positive selection.

**Table 1:**
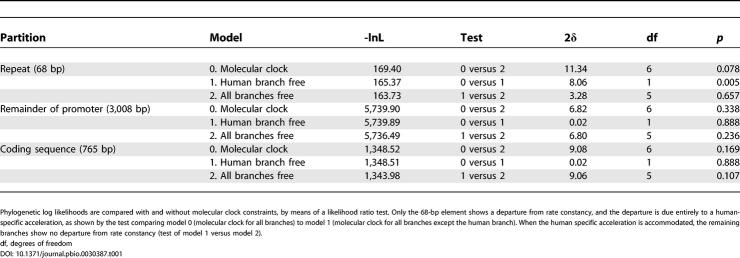
Molecular Clock Tests

**Table 2:**
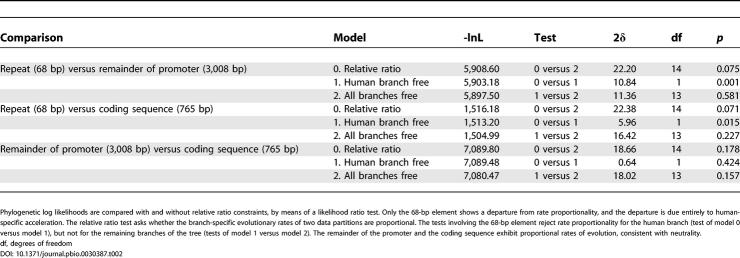
Relative Ratio Tests

The molecular evolution of the PDYN protein sequence, unlike the regulatory DNA, is consistent with a history dominated by negative selection. In a sample of the complete coding sequences from multiple chromosomes of eight primate species, 25 of the 254 amino acids in the PDYN protein vary, but none of the variants affect the 56 amino acids that comprise the neuroactive peptides. The exclusion of variation from the mature opioid peptides (Poisson *p* = 0.004) implies negative selection to maintain function. Phylogenetic likelihood ratio tests found no support for positive selection shaping the amino acid sequence of the remainder of the preprotein (model 1 versus model 2 of Yang et al. [47], *p* = 0.62). Nielsen et al. [13], in a genome scan of human-chimpanzee orthologs, also found no evidence for selection on the PDYN protein. No amino acid polymorphisms are known among humans, and we found none by directly sequencing the coding regions from chromosomes bearing each of the four repeat-number alleles of the promoter.

### Population Genetic Evidence: An Excess of High-Frequency-Derived Mutations Flanking the Selected Element

Positive selection alters the frequency spectrum of linked neutral mutations. As the selected mutations are driven rapidly to fixation, linked alleles are dragged along to high frequency [48]. The linked alleles may be dragged to fixation, but they may also be driven to high frequency and then decoupled from the selected mutation by recombination or allelic gene conversion. As a result, an excess of high-frequency-derived mutations flanking a fixed difference provides evidence for positive selection [49]. Our sample of 74 experimentally phased haplotypes from an Austrian population exhibits such a pattern (Table S1). Fay and Wu's *H* statistic is −8.13, strongly supporting a departure from neutrality and consistent with positive selection (*p* = 0.004). The three polymorphisms nearest to the 68-bp element have derived allele frequencies greater than 0.95 in all repeat-number allelic classes, consistent with a selective sweep that fixed mutations in the 68-bp region, and that thus predated the origin of different repeat alleles by tandem duplication. As the 68-bp element is tandemly repeated in all sampled human populations (Table 3), the signature of selection in all Austrian repeat-number allelic classes also implies that the selective events predate the global human diaspora. A sample of 20 chimpanzee haplotypes, though exhibiting many more polymorphic sites than the human haplotypes, and hence more power to detect a departure from neutrality, shows no such departure (*H* = 1.62).

**Table 3:**
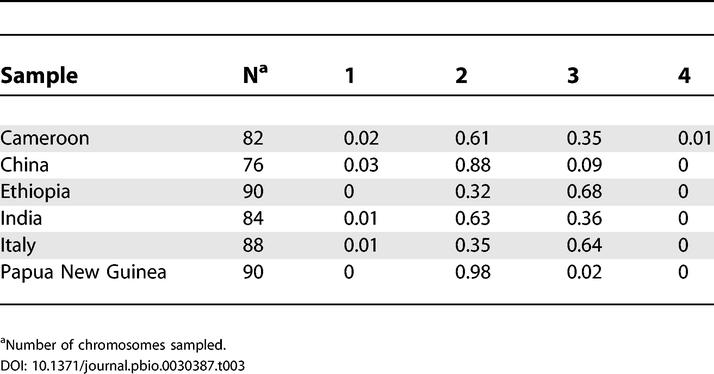
*PDYN* Repeat Allele Frequencies

The human-specific accelerated evolution of the 68-bp element is best explained as the result of positive selection favoring the fixation of mutations. Although the rate is elevated by a factor of more than ten over the neutral expectation, the selection intensity required to explain this excess is quite modest. The rate of substitution *(k)* is equal to the rate at which new mutations arise in the population (2*N_e_*μ) times the probability that a new mutation will become fixed [50], which is 1/2*N_e_* for neutral mutations and approximately 2*s* for advantageous mutations in a population of constant size [51], where *N_e_* is the effective population size, *μ* is the mutation rate, and *s* is the selective advantage of the mutant allele. If we let *f_a_* be the fraction of non-deleterious mutations in the 68-bp element that are advantageous, then the rate acceleration *(k_observed_/k_neutra_*_l_ *)* is the ratio of the substitution rate in the human 68-bp element ([1 − *f_a_*]*μ* + 4*N_e_sμf_a_*) to that expected in the absence of positive selection *(μ).* We can place bounds on *f_a_* by recognizing that there are only 204 base-substituting mutations possible in a 68-bp sequence. For the usual estimate [52] of long-term human effective population size, *N_e_* = 10,500, *s* falls in the range 0.0002 to 0.045; for *f_a_* greater than 2.2% (e.g., if all five fixed mutations were advantageous) *s* is less than 0.01 (Figure S1), well below the estimated selection coefficients of lactase persistence in Northern Europe [53] and G6PD deficiency [54] in regions of endemic malaria.

### Functional Evidence: The Selected Element Increases Inducible *PDYN* Expression

**Figure 2:**
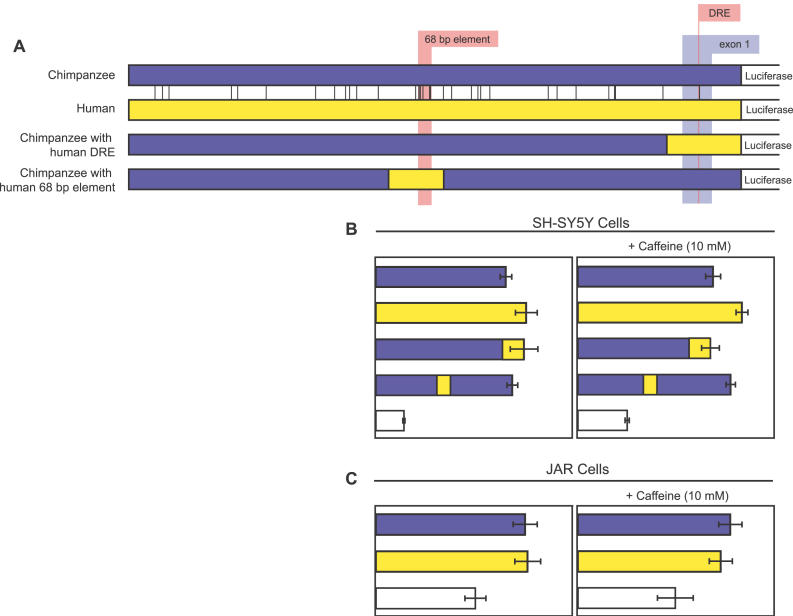
The Human 68-bp Element Increases Induced *PDYN* Expression We tested four 3-kb constructs, encompassing the region shown in Figure 1. (A) The human and chimpanzee constructs differ at the sites indicated by vertical bars. The two chimeric constructs incorporated the human 68-bp element or the human DRE (DREAM binding site) into the chimpanzee construct. (B and C) Panels show luciferase activity for each construct (± SEM, five to seven transfections), standardized to that observed for promoterless luciferase vectors (white bars), in SH-SY5Y and JAR cells, with and without added caffeine, which causes the release of intracellular Ca^2+^ and the release of DREAM from the DRE.

To determine the effect of the selected nucleotide substitutions on *PDYN* transcription, we transiently transfected the human neural cell line SH-SY5Y with constructs bearing 3 kb of human or chimpanzee *PDYN cis*-regulatory DNA linked to a luciferase reporter. Downstream of the 68-bp repeat, in the non-coding first exon of *PDYN*, is a downstream regulatory element (DRE), a binding site for the repressor protein DRE-antagonist modulator (DREAM) [55]. A single nucleotide substitution in humans alters the DRE from the sequence found in the other primate species. To isolate the effect of the substitutions in the 68-bp element from the effect of the substitution in the DRE, we generated chimeric constructs containing either the human DRE or the human 68-bp element in the context of the chimpanzee promoter (Figure 2).

We found that the human DRE sequence conferred slightly elevated expression of the reporter under basal conditions, though the effect was not significant (Figure 2B; analysis of variance *p* = 0.11). The sequence of the 68-bp element has no effect under these conditions (*p* = 0.66). When the effect of DREAM is removed by stimulating the cells to release intracellular Ca^2+^, which binds DREAM and causes it to release from DNA, the effect of the substitutions in the 68-bp element is conspicuous. Under these conditions, the human 68-bp element drives significantly higher expression than the chimpanzee sequence, regardless of the source of flanking sequence (Figure 2B; *p* = 0.002). Each relevant pairwise contrast is significant by *t*-test (human versus chimpanzee, 120%, *p* = 0.006; chimpanzee with human element versus chimpanzee, 115%, *p* = 0.037; human versus chimpanzee with human DRE, 120%, *p* = 0.007). In a three-factor analysis of variance, incorporating Ca^2+^ stimulation and the sequences of the DRE and the 68-bp element, the main effects of Ca^2+^ (*p* < 0.001) and the 68-bp element (*p* = 0.011) are significant and the interaction between Ca^2+^ and the 68-bp element is nearly significant (*p* = 0.054).

In contrast to the SH-SY5Y results, we observed no difference between chimpanzee and human constructs in the non-neural JAR cell line (Figure 2C), which serves as a control for the biological relevance of the *cis*-regulatory differences. Because *PDYN* is expressed in a broad range of neural and endocrine cell types and is induced by a diverse array of stimuli, our limited survey of potential functional consequences of human-specific regulatory substitutions is unlikely to have identified all such changes. Although transient transfection entails the removal of the regulatory DNA from its chromosomal context and the possible loss of biologically important interactions, the experimental results imply that the substitutions in the 68-bp element are visible to the cell.

### Continuing Selection: *PDYN* Exhibits Elevated Differentiation among Populations and Reduced Variation within Them

The evidence for positive selection on the functional 68-bp element, and hence for increased *PDYN* expression in humans, raises the possibility that selection has also acted more recently on the alleles that differ in the number of tandem repeats of the element following the origin of modern humans. Intraspecific *PDYN* variation is a plausible target for selection because variation in the number of repeats has been shown to affect inducibility by the phorbol ester TPA [36] and has been associated with protection against cocaine dependency [38] and with neurological disease [37,39]. Moreover, evidence for selection among human populations would corroborate the functional importance of the 68-bp element, and hence support the inference of selection in human origins.

Population genetics predicts that recent selection in human populations will leave two types of signatures in patterns of genetic variation: departures from neutral expectations in the pattern of differentiation among populations, and departures from neutral expectations in the pattern of variation within populations. These predictions have given rise to a battery of statistical tests: *F_ST_* -based tests to examine differentiation among populations, and *θ*-based tests to examine diversity within populations [56].

We initially genotyped the repeat polymorphism in six Old World populations and compared differentiation among populations (measured by *F_ST_*) at the repeat locus to the differentiation expected at loci evolving neutrally. Elevated *F_ST_* is a signature of geographically heterogeneous positive selection, driving allele frequencies to differ among populations more rapidly than they would if genetic drift and migration only were acting [57]. We estimated the neutral distribution of *F_ST_* values from a set of 18 mutually unlinked candidate neutral single nucleotide polymorphisms (SNPs) typed in the same individuals [58]. Each of the candidate neutral SNPs was selected for this preliminary screening on the basis of its high heterozygosity in Europe and its distance (more than 200 kb) from known genes. *F_ST_* values are constrained by the overall level of variation at a locus, so high heterozygosity is a useful filter for a pool of informative marker SNPs. Similarly, because genes and their regulatory elements are more likely to be under selection than arbitrary non-coding DNA, SNPs distant from genes are good candidates for neutral mutations.

**Figure 3:**
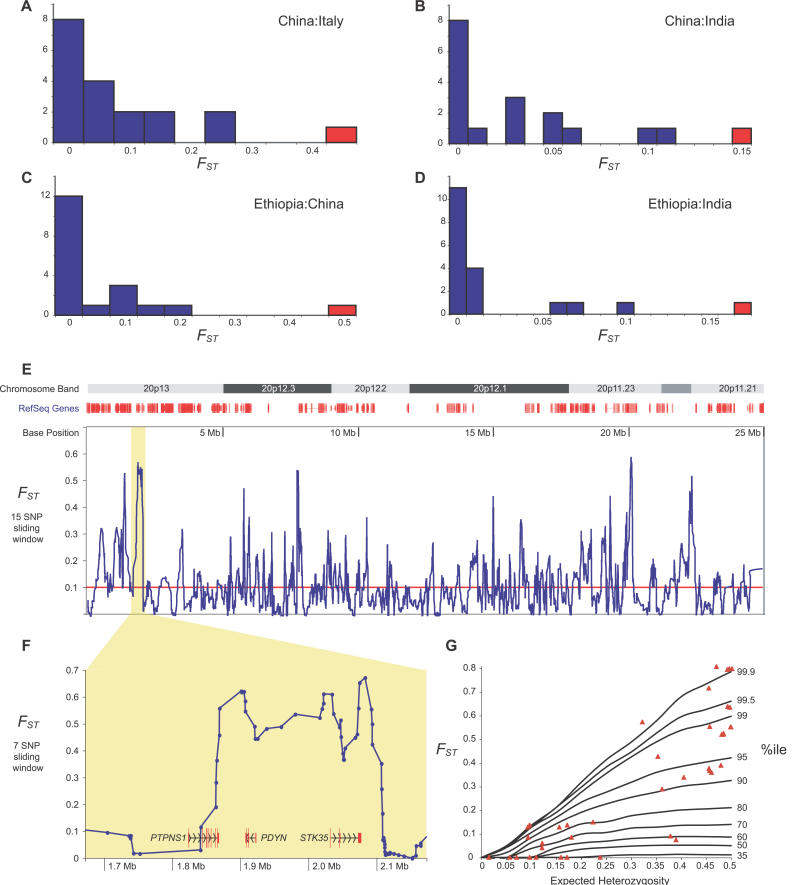
Elevated Differentiation at *PDYN* (A–D) In four pairwise comparisons, *F_ST_* at the *PDYN* 68-bp element (red) is markedly elevated above the *F_ST_* estimated from 18 candidate neutral markers (blue) typed in the same individuals. (E) Genetic differentiation between European- and Chinese-Americans, measured as a 15-SNP running *F_ST_* average, for the entire p-arm of Chromosome 20. *PDYN* falls under a large *F_ST_* peak (shaded), high above the arm average (red line). The RefSeq and chromosome band annotation is from the University of California, Santa Cruz Human Genome Browser (hg17), http://genome.ucsc.edu [79]. Perlegen SNP positions were matched to the hg17 assembly by the UCSC LiftOver utility. (F) A finer-scale sliding window analysis shows that the region of elevated *F_ST_* includes only two genes, *PDYN* and *STK35,* shown according to their RefSeq annotations. (G) *F_ST_* as a function of expected global heterozygosity. Red triangles represent the 52 SNPs in the Perlegen dataset in the 170-kb interval bounded by the 3′ ends of *PDYN* and *STK35.* The contours define the genome-wide density of *F_ST_* conditioned on heterozygosity; for each heterozygosity, the lines represent the *F_ST_* of SNPs in the specified *F_ST_* percentile.

Alleles with one or four copies of the *PDYN* repeat element are rare in every population we examined, but the frequencies of the two- and three-repeat alleles differ dramatically among populations (Table 3). The three-repeat allele ranges in frequency from less than 10% in China and New Guinea to more than 60% in Italy and Ethiopia. The differentiation at the repeat locus is higher than all 18 neutral markers for four of fifteen pairwise comparisons (Table 4), and the degree of elevation is substantial (Figure 3A-D). Although the small number of loci in our neutral proxy dataset makes it difficult to estimate precise significance values, we may approximate a denser probability distribution by bootstrapping over loci [58]. In this test, the difference between the *PDYN F_ST_* and the 18-locus estimate of *F_ST_* is significantly higher than the bootstrapped differences (*p* < 0.001) in the four comparisons. Moreover, *PDYN* has the second or third highest *F_ST_* in four more comparisons; the sum of *F_ST_* ranks across all 15 comparisons is significantly low (*p* = 0.01), although this *p*-value cannot be taken at face value due to the non-independence of the pairwise comparisons.

**Table 4:**
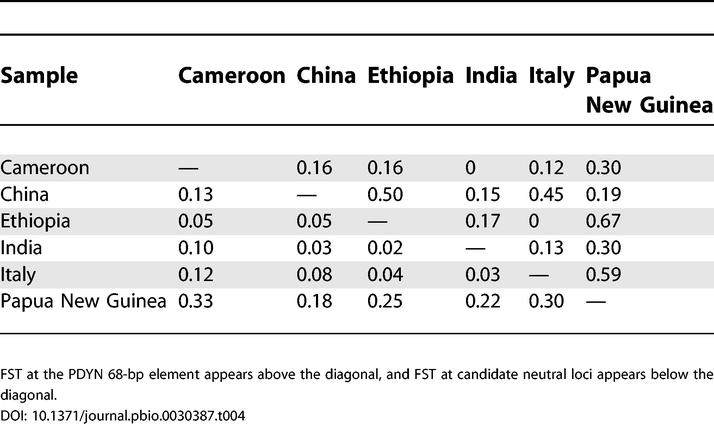
Pairwise *F_ST_* at *PDYN* and at Neutral Markers

If the elevated *F_ST_* at *PDYN* is due to positive selection favoring different alleles in different populations, the signature of selection should also be visible in nearby variants, whose evolutionary fates are tied to the selected variant by linkage. We therefore asked whether the *PDYN* locus falls within an extended region of elevated *F_ST_*. We investigated only Chinese-European *F_ST_*, the population contrast for which our data suggested elevated *F_ST_* (Figure 3A) and for which a genomic dataset was available. We used a dataset of 1,236,401 autosomal SNPs genotyped in African-, European-, and Chinese-Americans [59]. Because SNP ascertainment can influence the distribution of polymorphism statistics, we limited ourselves to SNPs ascertained by a single scheme: specifically, array-based resequencing of chromosomes from the National Institutes of Health Polymorphism Discovery Resource, a global sample. Because the 1.2 million SNPs share a common ascertainment bias, variation in *F_ST_* along the chromosomes will reflect only variation in the demographic and selective history of genomic regions.

As an initial screen, we generated a 15-SNP sliding window plot of *F_ST_,* considering only SNPs whose expected global heterozygosity exceeds 0.30. This filter is necessary to remove the dependence of *F_ST_* on heterozygosity; otherwise, the plot would primarily reflect variation in the allele frequencies of the genotyped SNPs rather than differentiation among populations. As Figure 3E shows, *PDYN* falls within a tall and broad peak in *F_ST_.* A finer scale sliding window plot (Figure 3F) indicates that the region of elevated *F_ST_* encompasses two genes, *PDYN* and a serine/threonine kinase *(STK35)* implicated in cytoskeletal regulation [60]. These genes are divergently transcribed, and their intergenic region therefore likely contains the majority of *cis-*regulatory DNA for both genes. The 3′ flanking regions of each gene also exhibit elevated *F_ST_.*

The genome-wide empirical distribution of *F_ST_* is shaped by both demography and selection, and therefore the tail probabilities of SNPs estimated from the empirical distribution represent a very conservative test for selection. Nevertheless, the SNPs within the *PDYN-STK35 F_ST_* peak exhibit significantly elevated *F_ST_*s*.* In Figure 3G, we plot *F_ST_* versus expected global heterozygosity for all 52 genotyped SNPs in the 170-kb interval defined by *PDYN* and *STK35* (i.e., excluding the 3′ flanking SNPs). We also plot the contours of the genome-wide *F_ST_* distribution conditioned on heterozygosity; note that the median *F_ST_* is below 0.06 for all heterozygosities. Six of the 52 SNPs in this region (12%) have *F_ST_*s in the top 0.5% of the genome-wide distribution, and 20 of the 52 (38%) are in the top 5%.

The number and location of selected variants driving elevation of *F_ST_* remain unclear. However, neither *PDYN* nor *STK35* is known to contain any non-synonymous variants, and neither protein sequence exhibits evidence of positive selection during human evolution [13]. The target or targets of selection are therefore likely to be *cis*-regulatory and to include the alleles of the 68-bp element.

**Figure 4:**
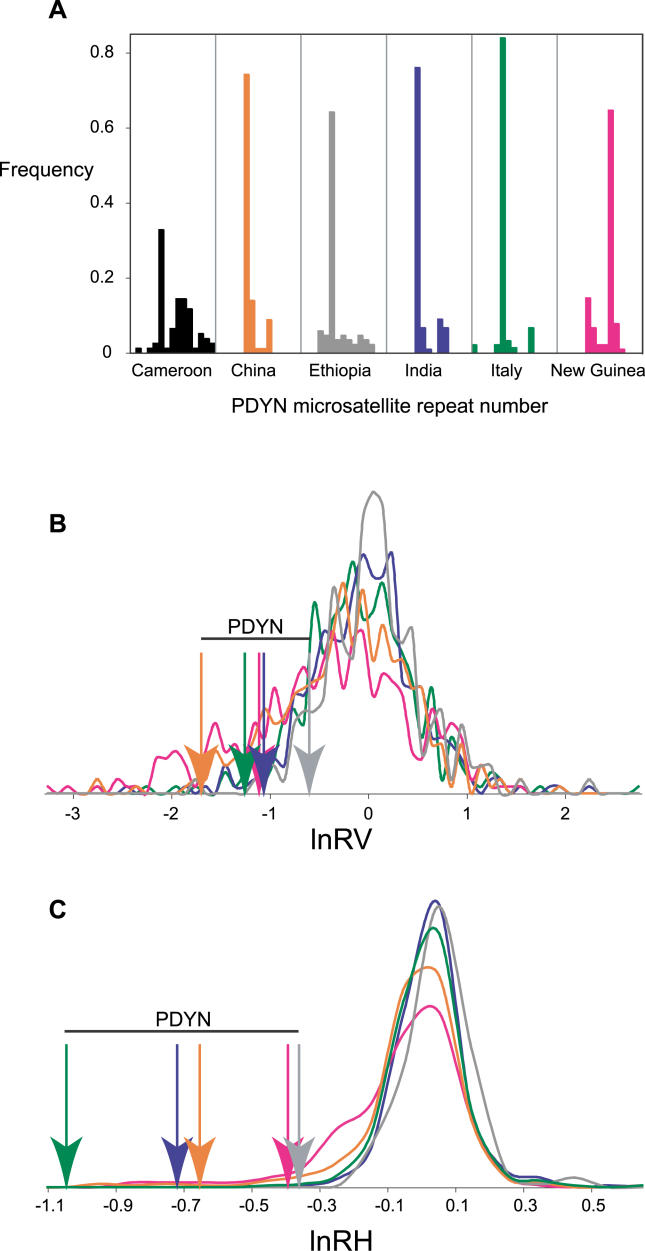
Altered Variation at the *PDYN* Microsatellite (A) The allele frequency distribution of the *PDYN* microsatellite for six populations. The most common allele has 18 CA repeats in each population except Papua New Guinea, where the 22-repeat allele is most common; the overall range is 13 to 27 repeats. The distributions show a reduction in allelic variation outside of the Cameroon population. (B) The empirical probability density of lnRV for a panel of genomically distributed microsatellites is plotted for each population, using panel A as the color key. The distributions are based on 193 microsatellite loci for Ethiopia and 377 loci for the other populations. For clarity, a single negative outlier from the New Guinea population has been omitted from the figure. The arrows indicate lnRV of the *PDYN* microsatellite for each population, in the left tails of the distributions, indicating a locus-specific reduction in repeat-number variance. (C) The empirical probability density for lnRH. Again, the *PDYN* microsatellite exhibits significantly negative lnRH values, indicating a locus-specific reduction in heterozygosity at *PDYN* in the non-West African populations.

Positive selection driving differentiation between populations should also decrease variation within populations; as a selected allele increases in frequency, its haplotype replaces other haplotypes before accumulating new variation. Microsatellites are particularly sensitive monitors of linked selection because of their high levels of polymorphism and high mutation rate. We asked whether the microsatellite nearest the *PDYN* promoter 68-bp element, a (CA)_13–27_ dinucleotide microsatellite 1.3 kb further upstream, exhibits the predicted signatures of selection. We genotyped the microsatellite in our panel of six populations (Figure 4A), and we used repeat-number variance and expected heterozygosity as summary statistics (Table 5).

**Table 5:**
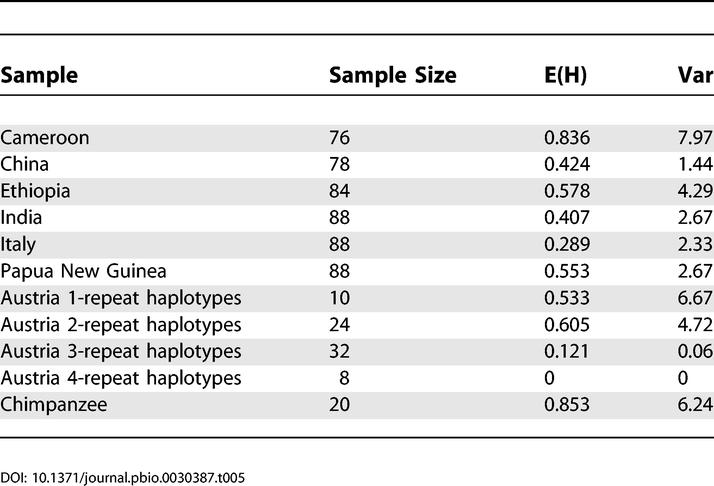
*PDYN* Microsatellite Summary Statistics

Repeat-number variance and heterozygosity are functions of *θ =* 4*N_e_μ* [61]. Because microsatellites vary in their mutation rates *(μ)* and recombinational contexts (which influences *N_e_*), we used test statistics that control for these effects. For a given microsatellite, mutation rate and recombinational context are expected to be shared among populations, so they cancel out in a ratio. The ratio, R*θ,* therefore estimates the relative effective sizes of two populations controlling for locus-specific phenomena; remaining variation among neutral microsatellites is attributable to stochastic variation in the outcomes of a neutral coalescent process [62,63]. Positive selection in one population will reduce heterozygosity and repeat-number variance at a linked microsatellite, causing it to appear in the tails of the estimated distributions of lnRV and lnRH (where repeat-number variance and heterozygosity are used in place of *θ*).

We estimated lnR*θ* distributions empirically from a genome-wide dataset of 337 autosomal loci [64, 65]. Because our *F_ST_* data do not indicate recent selection in the sample from Cameroon, we used Cameroon as the denominator in all ratios, and we tested for positive selection in the other populations. Those in which positive selection has acted are predicted to exhibit significantly negative lnR*θ* at the *PDYN* microsatellite*,* unless the Cameroon sample has experienced equal or more extreme positive selection at a *PDYN*-linked locus.

We found a significant reduction in repeat-number variance at the *PDYN* microsatellite (Figure 4B) in three populations (Italy, *p* = 0.031; India, *p* = 0.034; China, *p* = 0.021), but not in Ethiopia (*p* = 0.103) or Papua New Guinea (*p* = 0.209). The sum of lnRV ranks across populations places *PDYN* in the 2.5% tail of lowest sums among all the microsatellites. The reduction in heterozygosity at *PDYN* (Figure 4C) is even more extreme (*p* < 0.003 for Italy and India, *p* < 0.006 for Ethiopia, *p* = 0.016 for China, and *p* = 0.072 for Papua New Guinea). The *PDYN* microsatellite is the locus with the lowest lnRH rank summed over populations.

The relationship between the events reducing variation at the *PDYN* microsatellite and the events elevating *F_ST_* at the 68-bp repeat is most obvious when the haplotypic phase between the two elements is considered. We calculated expected heterozygosity and repeat-number variance in subsets of our experimentally determined haplotypes from an Austrian population. As shown in Table 5, the overall reduction in microsatellite heterozygosity and repeat-number variance is driven by the rapid elevation in frequency of the three-repeat allele at the 68-bp element.

The combination of elevated *F_ST_*s and reduced lnR*θ*s implies that the selection occurred in multiple populations, favoring the two-repeat allele in China and India, and the three-repeat allele in Italy and Ethiopia. However, it remains possible that the 68-bp element in the *PDYN* promoter is not itself the target of selection, as the entire *PDYN-STK35* region bears the signature of recent positive selection.

## Discussion

The phylogenetic and population genetic data described above are difficult to reconcile with a simple selective scenario. Instead, they point to a complex selective history at the human *PDYN* locus, with ancient positive selection acting across the species and more recent positive selection favoring different alleles in local contexts.

The data allow us to construct a coherent and plausible model that accounts for each observation. During the course of human descent from our last common ancestor with chimpanzees, multiple non-coding mutations arising upstream of the start of *PDYN* transcription altered the gene's *cis*-regulation and swept to fixation due to positive selection, as indicated by analysis of the primate sequence data and the elevated frequency of derived alleles flanking the fixations. Concurrently, mutations altering the neuroactive peptide products of *PDYN* were eliminated by negative selection. The proximate effect of the fixed *cis*-regulatory mutations is the upregulation of *PDYN* transcription, particularly when induced by intracellular calcium release. Subsequent to the selective sweeps, but prior to the peopling of the globe, the 68-bp region encompassing the fixed mutations duplicated. The timing of these events is supported by the presence of the fixed differences on all copies of the repeat, the high frequency of flanking derived mutations in all repeat-number allele classes, and the presence of the duplication in all sampled human populations. The duplication segregates today as a tandem repeat polymorphism, with one to four repeats. After the global human diaspora, human populations in different parts of the world experienced different regimes of selection on *PDYN cis*-regulation, as indicated by the elevated *F_ST_* values. Selection drove an increase in the frequency of the three-repeat allele in Europe and East Africa and independently increased the frequency of the two-repeat allele in India and China, according to the significantly reduced lnR*θ* values in each of the populations implicated by the *F_ST_* data.

The convergence of independent lines of evidence—phylogenetic, population genetic, and functional evidence for ancient selection, and evidence of recent selection in patterns of variation within and among modern human populations—underscores the importance of *PDYN* to human biology. Though *PDYN* has received little attention from human geneticists, our evolutionary genetic data suggest that the locus would repay further investigation. While we may point to possible environmental and cultural agents of recent selection, including differences in use of plant opiates and environmental inducers of endogenous opioids, such as acupuncture [66], the phenotype targeted by the ancient selection is unknown.

For thousands of years, people have used opiates to alter consciousness and ameliorate pain. Our data indicate that the evolution of our species involved changes in the inducibility of an endogenous opioid precursor, and that these changes were driven by positive natural selection. Changes in neuropeptide expression are known to have accompanied behavioral evolution in other species [67,68], but the difficulties in studying such changes in gene expression in living human brains have prevented their discovery until now. *PDYN*, a natural candidate for human-specific traits by virtue of its documented role in perception, emotion, nociception, and learning is the first documented instance of a neural gene whose *cis*-regulation has been shaped by positive selection during human origins. Although the transcriptional effects of the selected changes in the *PDYN* promoter appear to be subtle, slight changes in gene expression are capable of substantial effects on organismal phenotypes [69]. In keeping with the predictions of King and Wilson [21], our data imply that minor changes in gene regulation played a significant role in the evolution of the traits that make us human.

## Materials and Methods

### Cloning and sequencing

Our human haplotypes are from an anonymized collection of genomic DNA samples from an Austrian population [39]. To guarantee recovery of rare one- and four-repeat alleles, we selected DNAs of known repeat-number genotype (note that representative samples were chosen for population genetic analyses, described below). We PCR amplified 3-kb fragments of *PDYN* promoter from genomic DNA, using high-fidelity Phusion polymerase (Finnzymes, Espoo, Finland). For repeat-number heterozygotes, PCR products were cloned into pGL3-basic vector, using an invariant Acc65I site at the 5′ end of the promoter and a NheI site incorporated into the PCR primer at the 3′ end. For each haplotype, we completely sequenced multiple clones, and all singletons were verified by bidirectional direct sequencing. Repeat-number homozygotes were sequenced directly from PCR products; in cases of multiple-site heterozygosity, these PCR products were also cloned and multiple clones sequenced to determine phase. Non-human primate DNA was acquired from Coriell Repositories (Camden, New Jersey, United States) *(Pan troglodytes, Pan paniscus, Gorilla gorilla, Pongo pygmaeus, Macaca nemestrina,* and *Macaca mulatta),* and as gifts from A. Stone *(Pan troglodytes* and *Pan paniscus),* and D. Loisel *(Papio papio).* For each sample, Phusion PCR products were cloned and sequenced as above. We sequenced the two coding exons directly from PCR products. In addition to non-human primates, we included four Austrian samples with known promoter repeat genotype to ensure recovery of coding sequence linked to each repeat-number allele. Sequences were scored using Sequencher (GeneCodes Corporation, Ann Arbor, Michigan, United States).

### Phylogenetic and population genetic analyses

For all tests of substitution densities and rates, we conservatively assume that the site segregating among human repeat alleles represents a new mutation within humans and not a sixth fixed difference between humans and chimpanzees. To calculate the Poisson probability of five substitutions in 68 bp on the human branch, we found the expectation by considering the local average substitution rate, the genomic average divergence from chimpanzees, or the estimated genomic average mutation rate. The local average substitution rate, estimated from the human branch length for the entire 3-kb promoter sequence, including the 68-bp element, yields an expectation of 0.46 substitutions per 68 bp. At the broader scale of whole chromosomes, human and chimpanzee differ by nucleotide substitutions at an average of 1.44% of sites [43]; if one half of the divergence occurred on the human branch, the expected number of substitutions per 68 bp is 0.49. If instead of divergence, we consider the germline mutation rate, estimated at 0.99 × 10^−9^ per site, and assuming 5 to 7 million years of evolution since the last common ancestor of humans and chimpanzees [44], we expect 0.34 to 0.47 substitutions per 68 bp. Note that none of the substitutions occurs in a CpG context, although CpG may have been an intermediate in the adjacent substitutions that changed CpA in non-human primates to GpG in humans. The five substitutions are C→G, A→G, A→G, T→C, and C→A, three transitions and two transversions.

Molecular clock and relative ratio tests were implemented in HYPHY (http://www.hyphy.org) using an eight-sequence dataset, with a single allele representing each species. As our human exemplar we used the most common one-repeat haplotype from our sample of ten Austrian one-repeat alleles; we chose a one-repeat haplotype to facilitate comparison with the one-repeat sequences of non-human primates. The most common chimpanzee haplotype represented that species, while for other species, because each haplotype is unique in our small sample, a haplotype was selected randomly. Molecular clock tests used best-fit time reversible substitution models selected using ModelTest [70]. For the coding sequence and the promoter excluding the repeat, the favored model is HKY with Γ-distributed among-site rate variation. We used the maximum likelihood-estimated transition/transversion ratio and rate variation shape parameter and empirical base frequencies. For the repeat, the favored model is K2P. The relative ratio tests were performed using the HKY + Γ model, but results with K2P are very similar.

Negative selection on neuropeptides was tested by calculating the Poisson probability that zero of 25 variable positions would fall in the 56 of 254 amino acids comprising the neuropeptides. To test for positive selection in the remainder of the protein, we compared models 1 and 2 (*2δ* = 0.24364, *p* = 0.62) and models 7 and 8 (*2δ* = 0.0001, *p* = 0.99) from Yang et al. [47], in HYPHY, using the Goldman-Yang parameterization with base frequencies independent of codon position. The dataset included one sequence from each species.

For haplotype-based tests, we generated a representative population sample [71] by drawing 74 haplotypes from the sequenced Austrian haplotypes according to the population frequency of the different repeat-number alleles [36]. Summary statistics and their *p*-values were found using DNAsp [72].

### Intensity of selection

Estimation of the rate acceleration factor *(A)* involves three data partitions whose evolution is consistent with neutrality. First, we found the maximum likelihood estimate of the ratio of substitution rates between the 68-bp element and the remainder of the promoter, excluding the human lineage (ratio = 1.804). Next, we estimated the substitution rate on the human lineage for the portion of the promoter excluding the 68-bp element (rate = 0.00506 substitutions per site), holding the substitution model parameters constant. The product of these gives the expectation for the human 68-bp repeat under neutrality, 0.00913 substitutions per site. (Note that the Poisson probability of five substitutions, given that expectation, is 0.00005, and three or four mutations also fall in the 0.025 tail of the Poisson probability.) The maximum likelihood estimate of the substitution rate in the 68-bp element along the human lineage, 0.0926 substitutions per site, represents an acceleration factor *A =* 10.1. All estimates employed the HKY + Γ substitution model.

The genic selection coefficient *s* is estimated from the relations 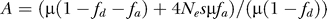 and *f*_*a*_ + *f*_*d*_ + *f*_0_ = 1, where *f_a_, f_d_,* and *f_0_* are the fraction of mutations that are advantageous, deleterious, and neutral, respectively. Figure S1 shows *s* as a function of the nuisance parameters *f_a_* and *f_d_.* We make the approximating assumption that *f_a_, f_d_,* and *f_0_* are constant over the course of the selective history of the locus.

To convert the acceleration factor to *s,* we consider the case of sequential fixations and ignore the effect of interference among independent advantageous mutations. The effect of interference is likely to be modest, as the expectation of the conditional fixation time of advantageous alleles, ~(2/*s*)(ln2*N*) [73], is less than 10,000 generations for *s* > 0.002, while the time available for fixations is roughly 300,000 generations (6 million years, 20 years per generation). Our estimate of *s* is based on the long-term effective population size since the divergence of humans and chimpanzees, which may be much larger than the estimate for modern humans. Larger *N_e_* translates into even lower estimates of *s*. The fixation probability (~2*s* for constant *N_e_*) is sensitive to fluctuations in effective population size [74], increasing during population expansions and decreasing during bottlenecks. Our simple approach assumes constant effective size.

The magnitude of the estimated rate acceleration excludes non-reciprocal exchange processes subsequent to duplication (e.g., gene conversion and unequal crossing-over) as possible explanations for the human-specific acceleration. Unbiased non-reciprocal exchanges do not alter substitution rates; although the number of sites available to mutate is increased by the number of repeat elements *(n),* the probability that a new mutation will spread among the repeats is 1/*n.* Biased processes can accelerate substitution [75], but only when the bias consistently favors new mutations over ancestral alleles. Even in the most extreme case, where every inter-repeat conversion event replaces an ancestral allele with a new mutation, the maximum rate acceleration is *n.* In human *PDYN, n* averages less than three and never exceeds four, and the long-term effective number of repeats (the harmonic mean of repeat number over the duration of the human lineage) is likely quite close to one. Strong bias is at any rate ruled out by the presence of a segregating variant among the repeats. So non-reciprocal exchange cannot produce the observed acceleration factor under neutrality. Under positive selection, however, tandem repeats, with or without biased conversion, can increase the power of deterministic forces relative to drift by increasing the effective population size to *N_e_n* [76].

### Vectors, cell culture, and transfection

We used the pGL3basic luciferase reporter (Promega, Madison, Wisconsin, United States). Chimpanzee and human constructs were generated as described above (“Cloning and sequencing”). To generate chimeric constructs with DRE site swaps, we cut the inserts with BstAPI and exchanged the DRE-containing fragments. To generate a 68-bp element chimera, we excised a human repeat using BspHI and DrdI and inserted it into a chimpanzee vector in the equivalent position. Vectors were verified by sequencing. We cultured JAR choriocarcinoma cells in RPMI 1640 with 2 mM L-glutamine and 10 mM HEPES, supplemented with 10% FBS. SH-SY5Y neuroblastoma cells were cultured in a 1:1 mixture of Ham's F-12 and EMEM with 1 mM sodium pyruvate and 0.1 mM non-essential amino acids, supplemented with 10% FBS. Both cell lines were acquired from ATCC and maintained at 37 °C with 5% CO_2_. We performed transfections in 24-well plates with JAR cells at 90% confluence and SH-SY5Y cells at 50% confluence. The transfection mix, in OPTI-MEM, included 2 μl of Lipofectamine2000, 0.72 μg of pGL3, and 0.08 μg of *Renilla*-TK (Promega) as a co-reporter to control for variation in transfection efficiency. At 26 h, medium was supplemented with growth medium with or without caffeine (final concentration 10 mM). Cells were harvested 16 h later. Luciferase activity was measured using the Dual-Luciferase Reporter Assay System (Promega) and a Turner Designs 20/20 luminometer. Results are reported as ratios of firefly:Renilla luciferase, standardized by setting the pGL3-basic ratio to one. Lysates from mock transfected cells were used to blank for machine background. All transfections were performed five to seven times, and effects assessed by analysis of variance and pairwise *t*-tests.

### *F_ST_* and lnR*θ* analysis

For the six-population analysis of *F_ST_,* neutral markers, DNA samples, *F_ST_* calculations, and bootstrap resampling are as previously described [58]. We genotyped the 68-bp repeat and the *PDYN* microsatellite by scoring the length of labeled PCR products run on an ABI 3700 capillary gel machine. We verified genotypes for 10% of samples by direct sequencing of PCR products.

Analysis of Perlegen data was limited to autosomal SNPs ascertained according to scheme A of Hinds et al. [59], array-based resequencing of National Institutes of Health Polymorphism Discovery Resource chromosomes. SNP data were downloaded from http://genome.perlegen.com/browser/download.html, and we used Perlegen's precalculated *F_ST_* values. To calculate expected global heterozygosity, we averaged the allele frequencies for the three genotyped populations and used 2*p*-2*p* ^2^ *,* where *p* is the average frequency of the global minor allele. To generate the percentile plot for *F_ST_* conditioned on expected heterozygosity, we sorted the SNPs into ten bins, each covering five percentage points of expected global heterozygosity range, we ranked SNPs within bins by *F_ST_,* and then we recovered the *F_ST_* for the SNP whose rank coincides with the relevant percentile within that bin. In Figure 3G, the contours are connecting the data points for the ten bins for each percentile; e.g., the point where the contours hit expected heterozygosity 0.5 represents the *F_ST_* percentile for SNPs with heterozygosities 0.45 to 0.5.

Microsatellite data were downloaded from N. Rosenberg's Web site, http://www.cmb.usc.edu/people/noahr/diversity.html, in *structure* format. The Rosenberg data lacked a population to match to our Ethiopian population, but data for 193 of the 377 loci were available from the dataset of Kayser et al. [65]. To represent our populations, we selected the populations in the Rosenberg data that are geographically coincident or proximate. As the distributions are quite similar for all populations (Figure 4B and 4C), the precision of population matching appears unimportant (also found by [65]). We selected as follows from the Rosenberg et al. data: Cameroon: Yoruba; China: Han Chinese; India: pooled samples from the populations included in Rosenberg et al.'s South Asia cluster, specifically Brahui, Balochi, Hazara, Makrani, Sindhi, Pathan, and Burusho; Italy: pooled samples from Sardinia, Tuscany, and Bergamo; Papua New Guinea: Papuan. For each microsatellite locus, expected heterozygosity was calculated as 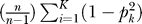 , where *n* is the number of chromosomes sampled, *K* is the number of alleles, and *p*_k_ is the frequency of the *k*th allele. Repeat-number variance was calculated as



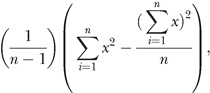



where *x* is the number of repeats in the *n*th chromosome.

The statistical properties of lnRV and lnRH allow us to estimate significance values in a parametric context, reducing the influence of any non-neutral outliers in the tails of the empirical distribution. Each of the five lnRV distributions is consistent with normality, according to a Kolmogorov-Smirnov test. Standardizing our observed test statistics according to the empirical mean and standard deviation, and using the tail probabilities of the standard normal distribution, we recover *p*-values nearly identical to those drawn from the empirical distribution (Italy: 0.034; India: 0.035; China: 0.016; Ethiopia: 0.119; Papua New Guinea: 0.236). Of the lnRH distributions, only the Ethiopian sample conforms to normality according to the Kolmogorov-Smirnov test, conferring a parametric *p*-value of 0.0002 on the Ethiopian *PDYN* microsatellite.

The causes of the departures of lnRH from normality are unclear, but ascertainment bias is an obvious possibility. The 377 microsatellites were ascertained in a European population and may be biased against microsatellites with low heterozygosity in Europe. In general, ascertainment bias is expected to be very modest for microsatellites because low-heterozygosity microsatellites are quite rare [62,64,77]. As Rosenberg et al. [64] note, their microsatellite data are very similar to data from microsatellites ascertained in independent, geographically diverse panels. Nevertheless, we must consider the possibility that ascertainment bias contributes to the shapes of the empirical lnRV and lnRH distributions. The expected effect of ascertainment bias is the truncation of the left tails of the distributions, due to the exclusion of loci with low heterozygosity in Europeans, and consequently the extension of the right tails. Two predictions are thus a departure from normality and a significantly positive skew, measured by the third moment about the mean. As noted, all lnRV distributions are consistent with normality and have well-behaved tail probabilities. Only the Ethiopian lnRV distribution has a significantly positive skew. For lnRH, four of five distributions fail the Kolmogorov-Smirnov test for normality, but the departures appear to be due largely to elevated kurtosis, not to positive skew. Only the Italian distribution has a significantly positive skew. We can construct a conservative test by drawing *p*-values from the right tails of the lnR*θ* distributions, which should be enlarged relative to the unbiased case; all lnR*θ* values are as extreme relative to the right tails as to the left, except for Ethiopian lnR*θ* values, whose *p*-values rise to 0.016 (lnRH) and 0.119 (lnRV). Because lnRH has a smaller coalescent variance than lnRV, lnRH is exquisitely sensitive to selection [63], and the observed departures from normality may therefore simply reflect the occurrence of selection at sites linked to some subset of the 377 loci [65,78]. The complete microsatellite dataset is presented in Table S2.

## Supporting Information

Figure S1 Intensity of Positive Selection The average selection coefficient *(s)* of advantageous mutations can be estimated from the rate acceleration of the human 68-bp element, conditioned on the fractions of all mutations that are advantageous, neutral, and deleterious. When the advantageous fraction is more than 2.5%, the average selection coefficient is less than 0.01. Over most of the parameter space, *s* is less than 0.001. The red line illustrates the case in which all and only the five fixed mutations are advantageous.
(1.7 MB EPS).

Table S1 Experimentally Determined *PDYN* Haplotypes from 74 Austrian Chromosomes Each unique haplotype is shown, from a sample of chromosomes selected to overrepresent the rare one- and four-repeat alleles. Derived alleles are highlighted in red. Haplotypes were determined by complete sequencing of multiple clones of each allele. Msat refers to the CA_n_ microsatellite 1.3 kb upstream of the 68-bp repeat. Position 2370 segregates TC_7_ and TC_9_ alleles. The ancestral states at Msat and 2370 are uncertain, as both sites vary among and within the other primate species.
(74 KB PDF).

Table S2 Microsatellite Summary Statistics For the *PDYN* microsatellite and those used to generate the genome-wide empirical distributions, we report the sample sizes, expected heterozygosities, and repeat-number variances, as well as the test statistics lnRH and lnRV.
(223 KB XLS).

### Accession Numbers

DNA sequences have been submitted to GenBank (http://www.ncbi.nlm.nih.gov.Genbank) with accession numbers AY902542–AY902679.

## Abbreviations

bp: base-pair
DRE: downstream regulatory element
DREAM: DRE-antagonist modulator
kb: kilobase
PDYN: prodynorphin
SNP: single nucleotide polymorphism
