# Supplementary material for: Ancient and Recent Positive Selection Transformed Opioid cis-Regulation in Humans
Source: PLoS Biol. 2005 Nov 15;3(12):e387. doi: 10.1371/journal.pbio.0030387 (PMC1283535; doi:10.1371/journal.pbio.0030387)
Supplement: Supplementary file 1 [file pbio.0030387.st001.pdf]

4rpt alleles (n=8) Haplotype Count	130	msat	530	550	780	810	1140	1240	repeats	1950	2120	2730	2940
4	T	18	C	T	G	T	C	C	GAGA	C	T	9	A
1	T	18	C	T	G	T	C	C	GGGA	C	T	9	A
2	T	18	C	T	G	T	C	C	AGAA	C	T	7	G
1	C	18	C	A	G	T	C	C	GGGG	C	G	9	G

3rpt alleles (n=32) Haplotype Count	130	msat	530	550	780	810	1140	1240	repeats	1950	2120	2730	2940
1	T	18	A	T	G	T	C	C	GGA	C	T	9	A
1	T	18	C	T	G	C	C	C	GGA	C	T	9	A
23	T	18	C	T	G	T	C	C	GGA	C	T	9	A
1	T	19	C	T	G	T	C	C	GGA	C	T	9	A
1	T	18	C	T	G	T	C	C	AAA	C	T	9	A
2	T	18	C	T	G	T	C	C	AAA	C	T	7	G
1	T	18	C	T	G	T	C	C	GAA	C	T	7	G
1	T	18	C	T	G	T	C	C	GGA	C	T	9	G
1	C	19	C	A	G	T	C	C	GGA	C	T	9	G

2rpt alleles (n=24) Haplotype Count	130	msat	530	550	780	810	1140	1240	repeats	1950	2120	2730	2940
3	T	18	C	T	G	T	C	C	GA	C	T	9	A
1	T	19	C	T	G	T	C	C	GG	C	T	9	A
1	T	18	C	T	G	T	C	C	GG	C	G	9	G
11	C	18	C	A	G	T	C	C	GG	C	G	9	G
1	C	19	C	A	G	T	C	C	GG	C	G	9	G
2	C	21	C	A	C	T	C	C	GG	C	T	7	G
1	C	22	C	A	C	T	C	C	GG	C	T	7	G
1	C	22	C	A	C	T	C	C	GG	C	T	9	G
2	C	23	C	A	C	T	C	C	GG	C	T	9	G
1	C	13	C	A	G	T	T	T	GG	T	T	7	G

1rpt alleles (n=10) Haplotype Count	130	msat	530	550	780	810	1140	1240	repeats	1950	2120	2730	2940
5	T	18	C	T	G	T	C	C	G	C	T	7	G
4	C	23	C	A	C	T	C	C	G	C	T	9	G
1	C	18	C	A	G	T	C	C	G	C	G	9	G

Table S1. Phased haplotypes.
Each unique haplotype is shown, from a sample of 74 Austrian chromosomes selected to overrepresent the rare 1- and 4-repeat alleles. Derived alleles are highlighted in red. Haplotypes were determined by complete sequencing of multiple clones of each allele. Msat refers to a (CA)n microsatellite, and position 2370 segregates (TC)7 and (TC)9 alleles; the ancestral states for these variants are uncertain, as both vary among and within other primate species.
